# Environmental factors induced crop raiding by wild Asian elephant (*Elephas maximus*) in the Eastern Economic Corridor, Thailand

**DOI:** 10.1038/s41598-023-40070-3

**Published:** 2023-08-17

**Authors:** Maneepailin Wettasin, Rattanawat Chaiyarat, Namphung Youngpoy, Nawee Jieychien, Ronglarp Sukmasuang, Phanwimol Tanhan

**Affiliations:** 1 Environmental Management and Technology, Faculty of Environment and Resource Studies, Mahidol University, Nakhon Pathom, 73170 Thailand; 2 Wildlife and Plant Research Center, Faculty of Environment and Resource Studies, Mahidol University, Nakhon Pathom, 73170 Thailand; 3 Department of Forest Biology, Faculty of Forestry, Kasetsart University, Bangkok, 10900 Thailand; 4 Department of Pharmacology, Faculty of Veterinary Medicine, Kasetsart University, Bangkok, 10900 Thailand

**Keywords:** Biodiversity, Conservation biology, Ecology, Zoology

## Abstract

Crop raiding are an increasing concern in wildlife conservation. This study identified the environmental factors that cause wild Asian elephants (*Elephas maximus*) to enter sub-urban and rural areas and share resources with humans in the Eastern Economic Corridor (EEC) in the eastern part of Thailand. The snowball method was used to interview villagers that had crop raiding experienced in seven provinces of the EEC and adjacent provinces in the eastern part of Thailand in 2020, and data from 183 households indicated that crop raiding had increased continuously from 2000 to 2020, especially in Chonburi, Chachoengsao, and Prachinburi provinces, which have seen increases in damaged agricultural areas. MaxEnt analysis showed an increase in incidents of crop raiding, while the elephants distribution area decreased from 9534 km^2^ in 2000 to 5199 km^2^ in 2010 and 4850 km^2^ in 2020. The study area has had land use changes in the low elevations from croplands of cassava and sugar cane to eucalyptus, para rubber, and fruits. These mixed crop plantations provide a pseudo-habitat for wild Asian elephants. The results from this study provide evidence that changes in land use and reduction of suitable habitat are factors that influenced the movement of wild Asian elephants to the rural agricultural areas and increased the incidents of crop raiding.

## Introduction

Crop raiding are a concern for elephant conservation. The wild Asian elephant (*Elephas maximus*) has been listed as an Endangered (EN) and their overall population decline has been driven in part by the expansion of agricultural areas and human settlements, habitat destruction, and hunting for tusks, meat, and skin^1^. However, in the lowland evergreen forest areas in the eastern part of Thailand, the populations of wild Asian elephant are increasing at an average annual rate of 8%, due to habitat suitability^2^, effective management, and greater accessibility to key resources owing to lower disturbance.

It is known that the deforestation of areas as a result of increasing human activities can lead to a decline in wildlife habitat quality and a decrease in natural food sources available to animals^3^. These phenomena lead to declining wildlife populations and habitat fragmentation^4^. Today, many countries have taken a responsibility to protect endangered species by establishing protected areas to recover wildlife populations. When habitat areas overlapping with human activities are not able to support their survival^5^, spatial and temporal overlap between elephants and humans can occur, leading to crop raiding and human–elephant conflict (HEC).

The species distribution models (SDMs) of elephants are important to understand the ecological of the animal and provided critical information for informed decision making^6,7^. Habitat is defined by a set of environmental factors under which a species can survive^8–10^. Various species distribution models are based on data measuring presence as well as absence of the species, for example Boyce et al.^11^, and Hirzel and Gwenaelle^12^. However, there is growing interest in modelling species distribution by using data consisting of observations of the presence of an organism at specific locations only^7,13–15^. This is called ‘presence-only’ data, because there is no reliable measurement of locations where the species was never present.

Presence-only models address the circumstance that presence only data is in most cases the only data available about a species. Absence data are not readily available for most species, especially for poorly sampled tropical regions where modelling potentially has the most value for conservation. In addition, even when absence data are available, they may be of questionable value in many situations as failure to observe a species is not necessarily equal to species absence. One spatially explicit species distribution model that is based on presence-only data is the maximum entropy (MaxEnt) technique. MaxEnt quantifies the ecological niche of a species translated in geographic space^16,17^. MaxEnt models probability of a species being present based on spatially defined variables, such as vegetation cover and land use, which might determine the habitat of that species. MaxEnt has consistently performed well in comparison to other methods^18^ and as presence data are common although they vary in accuracy^19^. The application of spatially explicit presence-only habitat models to real world situations has been shown to be useful within Asian ecosystems. The understanding of these relationships between spatially defined variables and habitat choice can help inform policy on conservation and sustainable utilization of biodiversity, by providing objective advice on human–elephant interaction, especially in frontier areas such as the Eastern Economic Corridor (EEC include: Chonburi, Chachoengsao, and Rayong) and adjacent provinces (include: Prachinburi, Chanthaburi, Trat, and Srakaew) in the eastern part of Thailand. EEC is an area-based development initiative, aiming to revitalize the well-known Eastern Seaboard where, for 30 years, numerous business developers have experienced a rewarding investment journey and exceptional achievements. The large agricultural areas in EEC that had high conflicted between human and wild Asian elephant will convert to industrial estates, and the habitat of wild Asian elephant will decrease. Therefore, models of wildlife habitat distribution are crucial for sustainable utilization and management strategies.

Today, wild Asian elephants often roam from their natural habitat to the forest patches outside protected areas because of the better water and food sources in these patches^20^. This phenomenon has been found in Khao Ang Rue Nai Wildlife Sanctuary (KARN) at the middle part of EEC. The water sources and artificial salt licks development and improvement by various agencies along the sanctuary boundaries may create more crop raiding such as rice, pipe apple, and sugar cane^21^. Furthermore, in the EEC, natural habitats have been impacted since 2016 by the Thai government policy to increase economic development and growth that includes the construction of infrastructure such as roads, high-speed railway, and power plants^22^. These projects involve significant land acquisitions that have impacted the homes and livelihoods of villagers^23^ and may contribute to increased HEC in the area.

There is a lack of research to understand the factors that cause wild Asian elephants to spread outside the protected areas. Therefore, the aim of this study was to identify the attitude of villagers that had crop raiding experiences as well as the factors leading to elephants wandering outside of protected areas and increasing the crop raiding in the EEC. The hypothesis of increased crop raiding was the extreme changed of land use in the EEC leading to elephants wandering outside of protected areas.

## Results

### Impacts of human–elephant conflict

**Figure 1:**
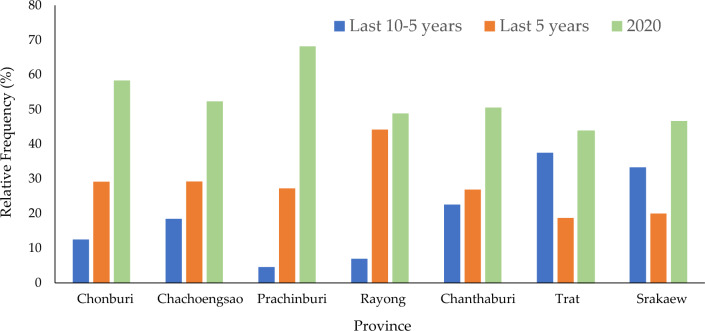
Human and elephant conflicts (HEC) in the eastern part of Thailand in the last 10 years (n = 183 households).

Form interviewed, 183 households (sex ratio was 6: 4 [males: females] and age structure was 20–40 years old [20%] and 40–60 years old [80%]) in seven provinces in the EEC in the eastern part of Thailand were differ in population size, remoteness, land use types, etc. In this study, a group of minor damaged on crops were not publicly known; however, local communities as the first group of samples were probably led to this group of samples in the provinces that are not well known in crop raiding. The results indicated that crop raiding were lower in the Srakaew, and Trat provinces, moderate in Rayong and Chanthaburi, and higher in Prachinburi, Chonburi, and Chachoengsao (F = 9.416, *df* = 2, 3, *p* = 0.001). Overall, crop raiding increased continuously from 2000 to 2010 and 2020 (F = 4.151, *df* = 2, 1, *p* = 0.042), and increases were especially higher in the higher crop raiding area (> 50% in 2020) and lower in the lower crop raiding area (Fig. 1). In 2020 and 2010, the results show that incidents of crop raiding in the EEC increased due to the land use and other environmental factor changes (Supplementary Fig. S1a–m and Table S1), especially in the higher crop raiding area.

**Figure 2:**
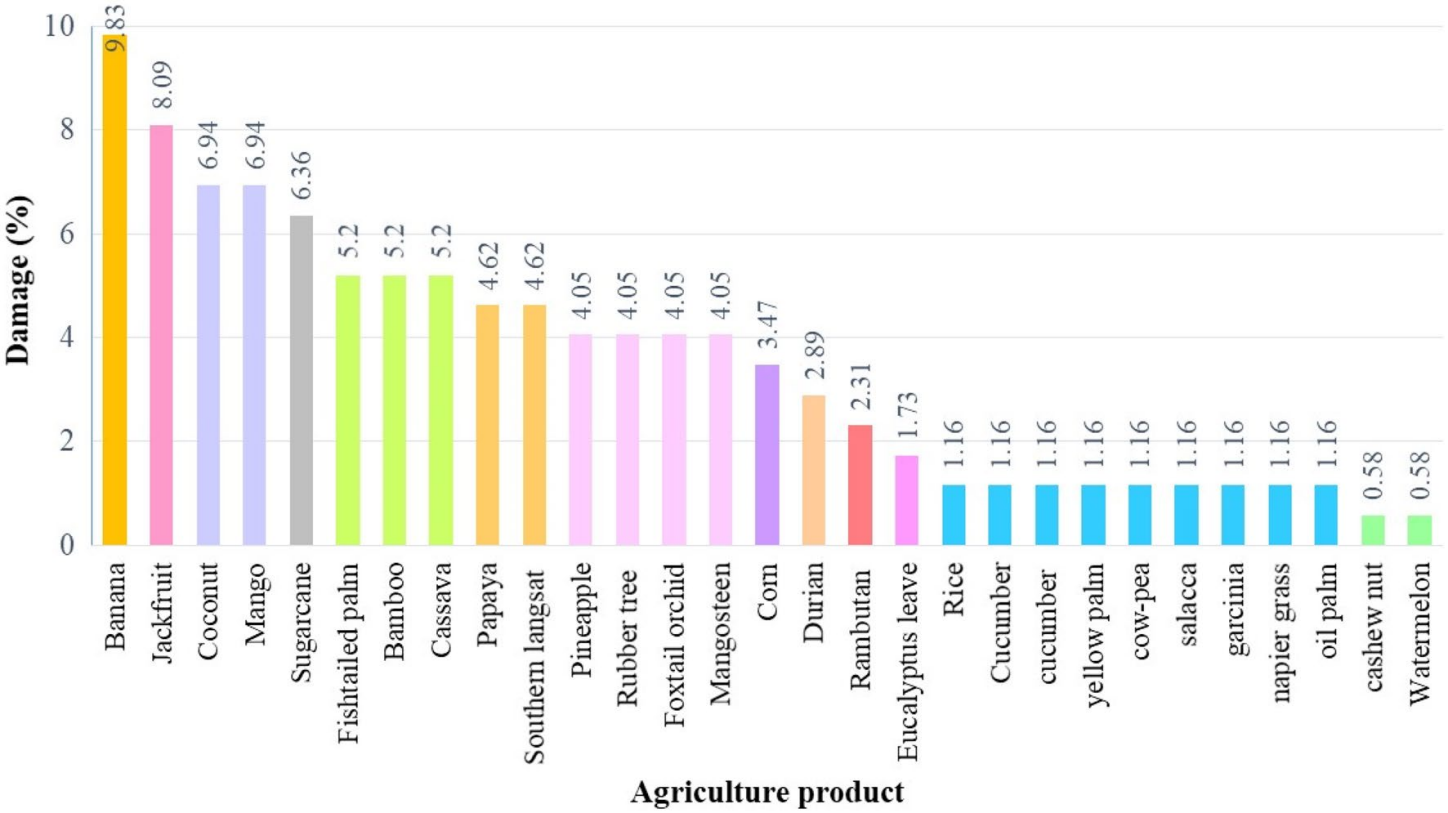
Consequences of crop raiding by wild Asian elephant in the eastern part of Thailand in the last 10 years (n = 183 households).

Examples of the consequences of crop raiding were damage to bananas (9.83), jackfruit (8.09), coconut (6.94) and mango (6.94), etc. (Fig. 2). The perception of villagers to coexist with elephants, they daily life were mostly, therefore, they were no comment about the attitude of coexist, and they can coexist with elephants in the area. While, in the higher crop raiding area such as Prachinburi province, the villagers with negative perception, they had attitude that they could not tolerate to elephants rather than they can coexist with elephants. The number of villagers killed by elephants in 2020 was > 10 people, and most deaths were found in the higher crop raiding area, especially in Rayong and Chantaburi provinces.

### Spatial distribution model

**Figure 3:**
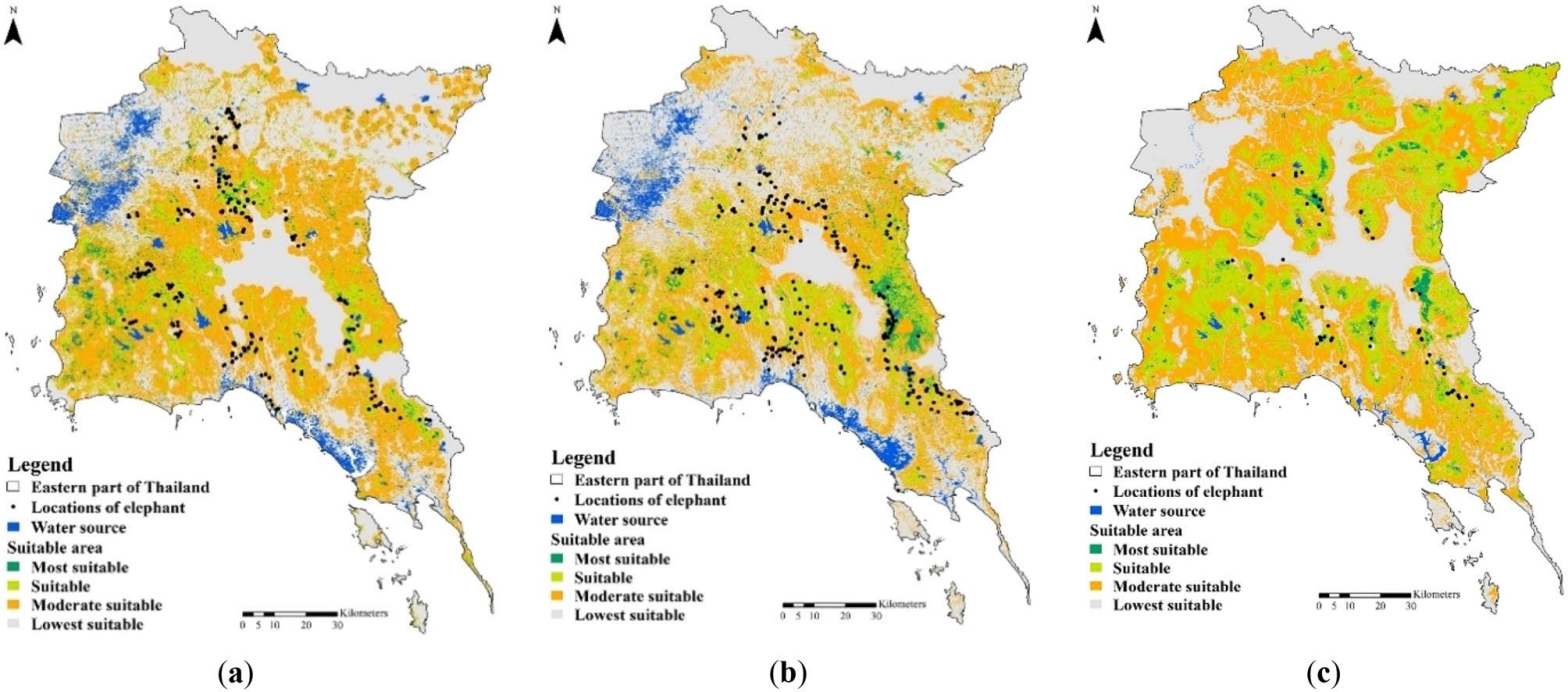
Three Species distribution Models of wild Asian elephant in the human settlement areas among (**a**) 2020, (**b**) 2010, and (**c**) 2000.

The category of wild Asian elephants habitat in the EEC and adjacent provinces was based on a 10 percentile training presence logistic threshold to determine the most suitable—lowest suitable habitat area of the wild Asian elephants (Fig. 3) due to the high level of land use changes in the EEC and adjacent provinces. The result of predicted habitat suitability (constrained to the eight variables used) from the Maxent model shows more suitable habitats are in the areas surrounded the EFC, and expanded between 2000 and 2020. In addition, the expanded of wild Asian elephant habitat may increase HEC in EEC in the future.

**Figure 4:**
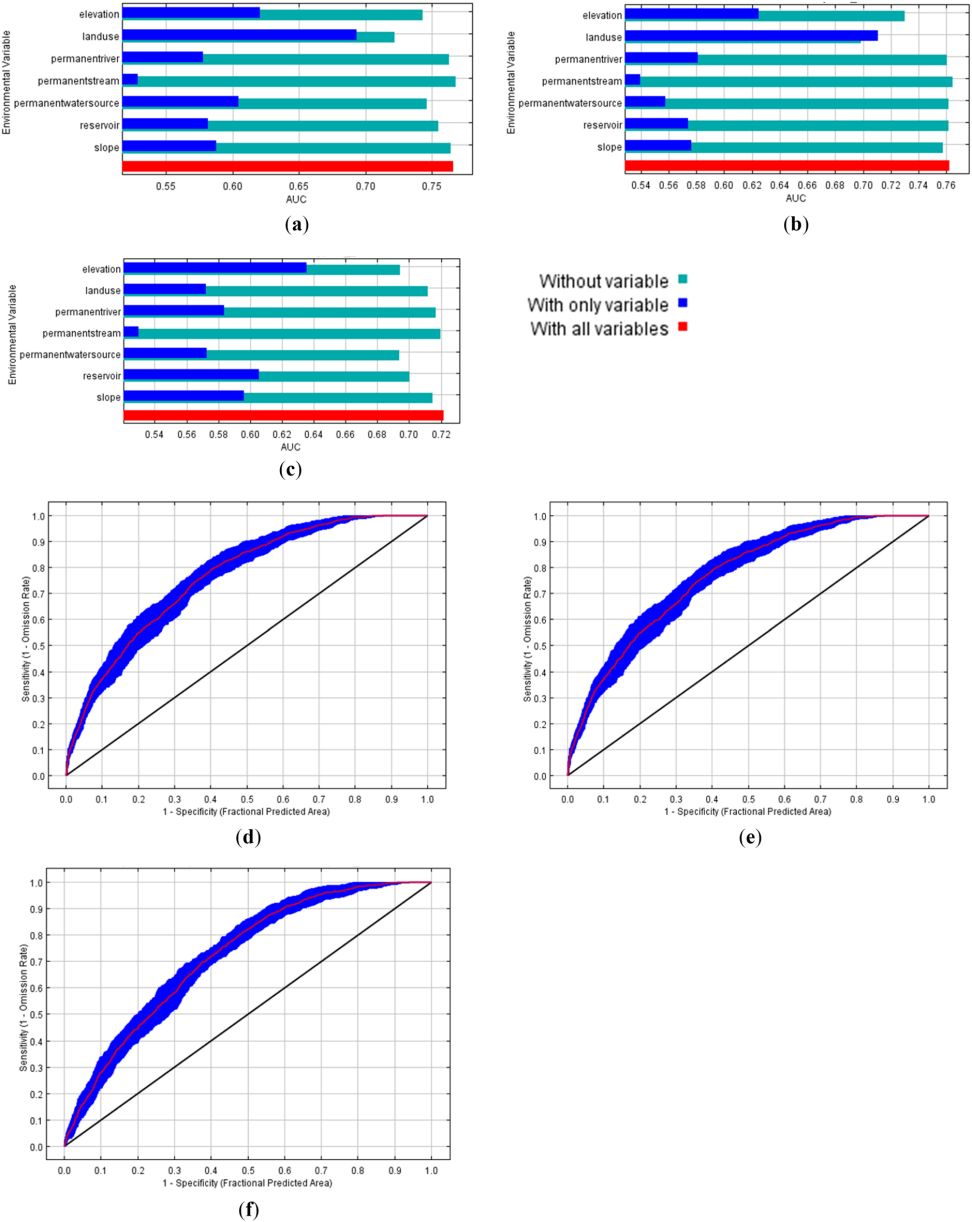
Curve of the receiver operating characteristic (ROC) curve and area under the ROC (AUC) for the wild Asian elephant habitat suitability model by using MaxEnt, the average test AUC for the replicate runs among (**a**) 2020 = 0.766, (**b**) 2010 = 0.762, and (**c**) 2000 = 0.722; and results of the jackknife test for contributions of the variables of the wild Asian elephant habitat model in the eastern part of Thailand in (**d**) 2020, (**e**) 2010, and (**f**) 2000. *Note*: Distance to permanence water sources (permanentwatersource, km), reservoirs (reservoir, km), land use types (landuse, km^2^), aquaculture farms (do not included in the figure, km^2^), permanence streams (permanentstream, km), temporary streams (do not included in the figure, km), permanent river (permanentriver, m), slopes (slope, %) and average sea level (elevation, asl, m).

In 2000, the SDMs was estimated 9534.7 km^2^ of most suitable habitat (AUC = 0.722) (Fig. 3a), and decreased to 5199.9 km^2^ in 2010 (AUC = 0.762) (Fig. 3b), and lowest in 2020 (4850.4 km^2^, AUC = 0.766) (Fig. 2c, Table 1). The forest plantations and agricultural areas located outside of protected areas were designated as most suitable for wild Asian elephants. The wild Asian elephant distribution in agricultural areas increased among 2000, 2010, and 2020 (Fig. 4a–f), while the habitat area in the protected areas were decreased due to land use changes (landuse) in 2010 (contribution > 70%, average AUC of SDM = 0.711) (Fig. 4b) and 2020 (contribution < 70%, average AUC of SDM = 0.694) (Fig. 4a) from agronomy such as pineapple, cassava, and sugarcane to eucalyptus and para rubber tree plantations as well as other fruit orchards in the low elevations (elevation) that were highest in 2000 (contribution > 60%, average AUC of SDM = 0.636) (Fig. 4c, Supplementary Table S2). These crop plants are cultivated in mosaics that are suitable for elephants as resting and sleeping paths in tree plantations such as eucalyptus and para rubber tree and feeding in agronomy such as pineapple, cassava, and sugarcane.

**Table 1:** Suitable area of wild Asian elephant in the eastern part of Thailand among 2020, 2010, and 2000.

Year	Area	Suitable area (km^2^)				
		Most	Suitable	Moderate	Lowest	Total
2020	EEC	138.5	1299.2	4054	5382.7	10,874.4
2020	Non-EEC	727.3	5932	18,583.3	24,336.8	49,579.4
2020	Total	865.8	7231.2	22,637.3	29,719.5	60,453.8
2010	EEC	82.1	1217.7	3382.4	6262.7	10,944.9
2010	Non-EEC	1199	6702	15,899.2	25,983.2	49,783.4
2010	Total	1281.1	7919.7	19,281.6	32,245.9	60,728.3
2000	EEC	136.2	2283.6	5129.3	3911.8	11,460.9
2000	Non-EEC	1135.7	13,480.9	19,138.7	18,392	52,147.3
2000	Total	1271.9	15,764.5	24,268	22,303.8	63,608.2

Eastern Economic Corridor (EEC include: Chonburi, Chachoengsao, and Rayong) and non-EEC: include: Prachinburi, Chanthaburi, Trat, and Srakaew.

From the survey results, some villagers expressed an inconvenience in living together with wild Asian elephants. In the areas with high risks of property loss and personal injury, the villagers preferred to move all wild Asian elephants to the protected areas as were seen in the past and require reproduction control of these elephants. Some villagers preferred that the government setup a wild Asian elephant sanctuary and change the behavior of these wild elephants to reduce crop raiding.

## Discussion

These crop raiding experiences is the special group that was difficult to access subjects with the target characteristics. Snowball sampling method is good to find the subjects recruit future subjects among their acquaintances, and the sampling continues until data saturation^24^, and can reduce a bias among the seven provinces in EEC and non-EEC, when the sampling is used where potential participants are hard to locate^25^. Even though, this method is well suited for this study and a number of research purposes^26^ and is particularly applicable when the focus of study is on a subtle issue, possibly concerning a relatively secretive matter, and thus requires the knowledge of insiders to locate people for the study and it is impossible to determine the sampling error or make inference about the population on the samples obtained^25^.

The changed in land uses are the main focus for development of industrial estates^22^ in the lowland habitats (low elevation and slope)^27^ that were suitable for wild Asian elephant in 2000. These changes in land use can created large open areas and fragments in the eastern part of Thailand, resulting in habitat mosaic^28^ that were the suitable habitats for wild Asian elephants. Thus, these phenomena led to crop raiding due to the wild elephant’s preference for lowland habitats^29^ and certain types of crops in this area^2^. There have been trends of preferring dry evergreen forest and forest plantation for resting and sleeping, and using crop plants such as pineapple, cassava, and sugarcane for feeding as found in Myanmar^29^ and monoculture trees such as acacia, eucalyptus, abandoned coffee, and reserved forest remnants^30^.

The decline in natural forest habitats and an increase in human activities in the area have led to more incidents of crop raiding as found in the Kaeng Krachan National Park to surrounding land use^31^, and the Aceh province, where that the main factor causing crop raiding was distance to the human settlement^32^. The landscape level evaluation of discrete elephant populations in these regions seems to be of paramount importance, as habitat loss and loss of connectivity seems to be poised to extinguish smaller isolated elephant populations in this region over time as found in Western Forest Complex of Thailand^33^, exacerbated by loss of lowland habitats and changes in semi-useful agricultural crops. The characterization of crop raiding over time and space as well as less tolerance for elephants are associated with fragmentation of elephant habitats and populations as well as loss of favorable habitats over time due to agricultural expansion and changes^33,34^. In the future, crop raiding may not only involve damaged fences, orchids, para rubber leaves, eucalyptus, bananas, cashew nuts, and other fruits but also become more serious when the lands become industrial estates and urban communities, as found in China^5^. This problem will impact to the habitat and food dynamics of wild Asian elephants, and may lead to increases in crop raiding in the overlapping areas^35^ due to an increase in wild elephant population in the protected areas^28^ and movement into more favorable habitats^34^ as the natural habitat is decreases.

The SDMs showed that low elevation was important in the most suitable and suitable areas for wild Asian elephants in the EEC. The wild Asian elephants generally avoid feeding or walking in upland areas in order to save energy^36^. The Sumatran elephants preferred the areas with lower elevation and 0–20% slope; the elevation was < 200 m in Indonesia^37^. Wild Asian elephants in the eastern part of Thailand mainly used shallow slopes (0–20%), a finding similar to those from other studies^38^. Most of the wild Asian elephants were found in the flat plains, consistent with the findings from other studies^39^. However, the finding this group of wild Asian elephants differs from that in wild Asian elephants that live on the top of a plateau in the Phu Kieo Wildlife Sanctuary that is close to 1000 m above average sea level^40^. Waterholes are not a main environmental factor in the eastern part of Thailand, as reported by Alfred et al.^39^, since water is found in a majority of the area. However, the water resources in some protected areas are increasing as agricultural needs and climate change, and may be increase the crop raiding on agricultural areas in surrounding communities^41^.

After the year 2000, many reservoirs were constructed to serve the EEC and there are plans to increase the number of dams and reservoirs in the area^36^, which will decrease terrestrial habitat^42^ and create large water bodies. The land areas around these water sources will become human settlements, and agricultural areas and other human activities that will decrease the availability of water throughout the area that elephants can access. A large section of the EEC is covered with evergreen forests, proving to be the most suitable habitat for wild Asian elephants, in contrast with the Salakphra Wildlife Sanctuary where the most suitable habitat is mixed deciduous forests since bamboo, a favorite food of wild Asian elephants, is dominant in the area^38^.

In 2010 and 2020, the land use change was associated with habitat utilization of wild Asian elephants in the EEC. Large areas of food-plant production have been found to be positively related to utilization by the species, and crop plants such as cassava and sugarcane have been reduced and replaced with tree plantations such as eucalyptus, para rubber trees, and oil palms^30^. The increasing number of plantations has reduced the suitable areas for the wild Asian elephants, as found in this study, while the frequency of wild Asian elephant movement outside the protected areas has led to dramatic increases in crop raiding^43^ that increased in 2020. These are primary factors that have been previously reported to affect their movements^44^ and population dynamics^40^, and as a result, wild Asian elephant movements are known to be strongly controlled by water availability, especially during the dry season^45^.

The villagers reported some inconvenience in living together with wild Asian elephants in the EEC as they are the roots of crop raiding. In the areas at high risk of danger to lives and property loss^46^, the villagers preferred to move all wild Asian elephants to the protected areas as was done in the past. Some villagers preferred that the government setup an elephant sanctuary to keep the wild Asian elephants in the areas and to change the behavior of these wild elephants^47^. These strong recommendations of people increased when they were not satisfied with the present conflict management strategies due to their ineffectiveness as found in Nepal^48^.

The lower crop raiding area such as the Srakaew, and Trat provinces, the villagers had high perception to supported the attitude of human can coexist with wild Asian elephants, while in the higher crop raiding area such as Chonburi, Chachoengsao, and Prachinburi provinces, the perception not to be tolerant with wild Asian elephants increased when crop raiding negatively impacted the local social economy^49^. In the higher crop raiding area, especially in Rayong province, the villagers had perception that they can or cannot coexist with elephants was equally as high as the number of deaths, and our finding was similar to that of Kinyanjui et al.^50^, who suggested that unsustainable income, food insecurity, and human–human tensions play an important role in influencing risk perceptions and increasing perceived conflicts with elephants. The increasingly vulnerable population in these communities may shift their perceptions toward one of intolerance and conflict with elephants in the future. Conversely, Van de Water & Matteson^46^ reported that those who had received benefits from living near elephants had more supportive views of elephant coexistence. Plantation owners reported using a variety of deterrence methods with varying success, with firecrackers being the most commonly utilized method. Community members identified several potentially beneficial mitigation strategies including forest restorations and patrol teams, adding water sources to wild elephant habitat, and education of local school and community groups.

Our study suggests that the distribution of wild Asian elephants in the eastern part of Thailand is influenced by altitude and land use change. The most suitable areas for the existence of elephants and the least useful ones were fluctuations in the years 2000, 2010, and 2020 in term of area. Furthermore, the trend for the expansion of wild Asian elephant habitat use areas in the eastern part of Thailand is expected to increase due to the high land use change. Also, a reduction in suitable habitat are factors that influenced the movement of wild Asian elephants to the sub-urban and rural agricultural areas and increased in incidents of crop raiding.

In the future, the monitoring the population trends, food quality and quantity as well as the body condition of this population is required for conservation and management and to ensure the long-term conservation of the area. Furthermore, regular monitoring and surveys are required to build up a comprehensive database on the population trends and controlling, improve public awareness, and combined with habitat management, this may help reduce crop raiding in the area.

## Methods

### Study areas

The eastern part of Thailand (UTM 800,000 N, 50,000 E) has hilly and mountainous areas characterized by mountain ranges, with the highest peak being the Khao Soi Dao Mountain at 1675 m above sea level. Over 30% of the terrain is sloped with many streams and waterways, creating undulations, and the foothills have a constant slope of less than 30% (Fig. 4)^51^. Most of the areas are covered with dry evergreen forest, and there are numerous streams from the mountains. Coalescing fan hills are formed close to the mountain ranges due to aggregation of sediments carried in streams on the foothills. The low terraces were once watershed areas that are dry due to the reduction in river water levels.

The Northeasterly monsoon winds and the Southwesterly monsoon create two main seasons; the dry season (November to April), and the wet seasons (May to October). The average temperature is 26.8 °C; the average highest temperature is 31.6 °C, mostly occurring in April. The average lowest temperature is 23.3 °C. The relative humidity is influenced by the ocean and is high year-round, averaging 80%, while the average annual precipitation is 2874 mm due to rainfall year-round^51^.

The forests are rich in plant biodiversity owing to the year-round rainfall and the numerous sources of numerous streams. Important species include *Hopea odorata*, *Afzelis xylocarpa*, *Tetrameles nudiflora* and *Irvingia malayana*. Plants in the dense understory include *Calamus* spp., and *Amomun villosum*. In disturbed areas, the deciduous forests contain grasslands and plant species such as *Shorea obtuse*, *S. siamensis*, *Dipterocarpus tuberculatus*, and *Xylia xylocarpa*^52^ that feed animals.

Important mammals in the area include wild Asian elephants, gaurs (*Bos gaurus*) and bantengs (*B. javanicus*). Tigers (*Panthera tigris*) were the largest predator before their extinction in the area. The KSD is home to endemic extremely rare species such as the silver pheasant (*Lophura nycthemera lewisi*). Crop plants include para rubber tree, eucalyptus tree, various fruit trees, oil palms, cassava, corn, sugar cane, papaya, and rice^52^ (Fig. 3).

Wild Asian elephants are distributed in all seven protected areas in the Eastern Forest Complex (EFC): KARN, Khlong Khruea Wai Chaloem Phra Kiat Wildlife Sanctuary (KKWC), Khao Soi Dao Wildlife Sanctuary (KSO), Khao Chamao-Khao Wong National Park (KCKW), Khao Sip Ha Chan National Park (KSHC), Khao Khitchakut National Park (KK), and Klong Kaew National Park (KKN)^2^. The number of wild Asian elephants in the study area, living both in and out of protected areas, was estimated at 334 individuals in 2015^2^. The wild Asian elephant population located in each protected area was 206 individuals in 2015^2^ and 80–334 individuals in 2018^21^ in KARN, 45 individuals in KSD, 43 individuals in KCKW, 23 individuals in KSHC, 15 individuals in KKWC, one individual in KK, and one individual in KKN^2^. The wild Asian elephant density of KARN was 0.9 individuals/km^2^ in 2019^21^. Furthermore, many wild Asian elephant herds were roamed freely near the edge of protected areas, and were active from 6 pm until sunrise at 6 am of the next day to avoid heat during the daytime^21,53^. However, they were most active between 7:30 and 10:00 pm^2^.

### Impacts of wild Asian elephant in agricultural areas

In this study, snowball sampling was used to find subjects with the target characteristics^24^. The initial study subjects recruited additional subjects from their acquaintances. Thus, this method is efficient and cost-effective for accessing people that had crop raiding experienced who would otherwise be very difficult to find. In this method, the existing study subjects recruit future subjects among their acquaintances, and the sampling continues until data saturation^54^. Snowball sampling may be less reliant on a reference sample, although it is still suitable for finding unattainable populations. For instance, a group of minor damaged on crops were not publicly known; however, local communities as the first group of samples were probably led to this group of samples. Snowball is appropriated with the Maximum Entropy (MaxEnt) model that uses presence-only data and is designed as a general-purpose SDM^16^, and has been shown to perform well with small datasets^55^. Okello et al.^56^ used this technique to interview 78 people in Amboseli area which is over 5000 km^2^ with surrounding Maasai group ranches and privately owned lands, the Kenya Wildlife Sanctuary local leadership (political, administrative, and group ranch leadership of the local community) and selected people knowledgeable and involved in elephant conservation in the Kenya Wildlife Sanctuary, research and conservation organizations to study the prevalence and severity of current HEC in Amboseli Ecosystem, Kenya. Additionally, anonymity and confidentiality of the data can be guaranteed by the researcher^57^.

The questionnaire focused mainly on the location of different incidents of crop raiding found in seven provinces in the EEC among damaged areas in 2000, 2010 and 2020. The attitudes towards wild Asian elephants in general, with some questions focusing specifically on the villagers respondents’ attitudes toward coexisting with wild Asian elephants as used by Gangaas et al.^58^ to study the geo-spatial aspects of acceptance of illegal hunting of large carnivores in Norway (Country n = 18, Municipality n = 429) and Sweden (Country n = 19, Municipality n = 280), Scandinavia. The 183 interviews with HEC experiences were collected in seven provinces in the eastern part of Thailand to ensure that the survey covered the range of environmental factors. These locations and environmental factors would later be used to analyze the SDMs^26^.

The number of coordinate locations in this study was close to 195 interviews of agro-pastoralists by Drake-Brockman^59^ and 130 interviews in Somaliland covers an area approximately 137,600 km^2^ by Evangelista et al.^26^. After the interview, the villagers were shown the location of elephants entering the communities, and then the locations were observed and recorded by using GPS. For validity and reliability, we triangulated observation and interview-derived data across researchers, comparing notes and impressions. In particular, author N.C. participated simultaneously as a volunteer in elephant chasing teams. These different roles, independently and jointly, were essential in developing a profound understanding of the villagers’ thoughts. Furthermore, combining interviews with observations allowed the researchers to gain high-quality data and insight into a notoriously difficult-to-observe phenomenon, 2255 km^2^ located in the northeasternmost part of the Piedmont region, close to the border of Switzerland, in the central Italian Alps^60^.

**Figure 5:**
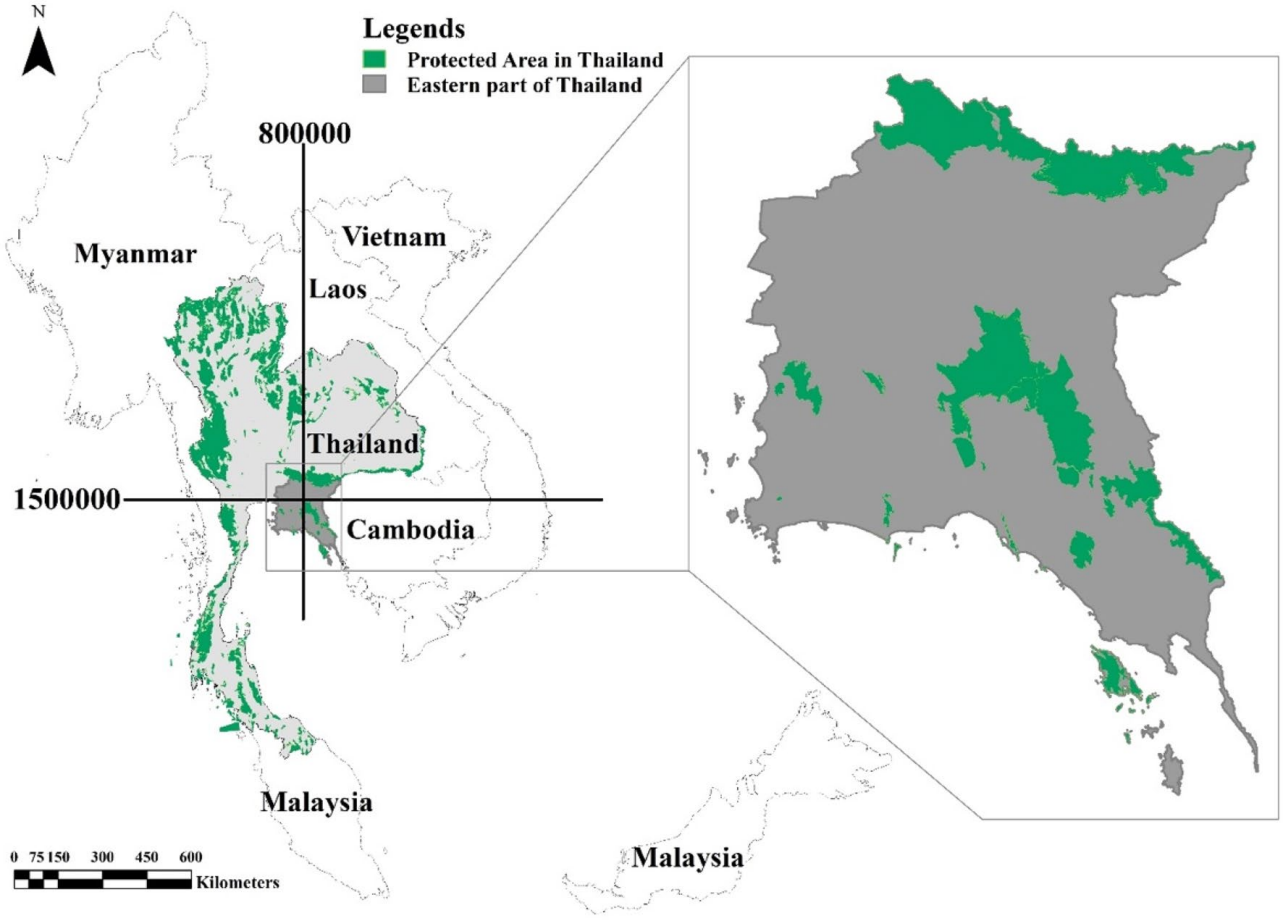
Location of protected areas in the eastern part of Thailand.

Interviews were semi-structured to obtain the information needed and also to maintain flexibility on aspects relevant to the interviewee^61^. The interviews were started with households in the Chachoengsao province whose crop plants had been damaged by elephants the previous night, and which had claimed damages to the head of a village. This information on crop raiding were send to a local office and civil defense volunteer, and the records were used to identify the first subject to initiate the snowball process. The Chachoengsao province had the highest number of impacts in the EEC^21^. In the eastern part of Thailand, the main protected areas in EFC are located at the center, while the villages are located along the boundary of the EFC (Fig. 5). As the interviews proceeded, respondents provided the names of new potential interviewees in the community, which in turn led to further opportunities for data collection. The second interviewee was suggested counter clockwise to the EFC, and the interviews were continued in this direction and stopped when the last interviewee was in the subdistrict with the first interviewee. This iterative exercise yielded direct and participant observations of 302 households in 2020; the most relevant excerpts of which are reported here in our own translation. The sex/age distribution, damage caused by wild elephants to agriculture in general as well as other impacts to properties and human wellbeing such as death or injuries and attitude of villagers on living together with wild elephants in the EEC and the adjacent provinces in the eastern part of Thailand were interviewed and recorded for analysis. The coordination of the damaged areas was record by using handheld GPS. This method can be applied in the large-scale, cross-sectional data collected from subjects using a web-based survey recruited through convenience snowball sampling^62^.

### Species distribution model (SDMs)

Species distribution models are widely used in cases where wildlife is constantly moving and cannot be counted directly^63^. Previously, SDMs employed geographic information systems to analyze spatial data and parameter-based statistical analysis and classify suitable habitat models^1^ using presence-absence data and presence-only data.

The MaxEnt is a general-purpose method for making predictions or inferences from incomplete information, and its origins lie in statistical mechanics^64^. The objective of the MaxEnt is to estimate a target probability distribution by finding the probability distribution of maximum entropy, and is subject to a set of constraints that represent the incomplete information about the target distribution^65^. The information available about the target distribution often presents itself as a set of real valued variables, called features, and the constraints are that the expected value of each feature should match its empirical average. All locations of wild Asian elephants in the seven provinces were analyzed using SDMs with environmental factors in 2000, 2010, and 2020 such as distance to permanent water sources (km), distance to reservoirs (km), distance to land use types (km), distance to aquaculture farms (km), distance to permanent streams (km), distance to temporary streams (km), slopes (%), and altitude above sea level (asl, m) (Table 2 and Supplementary Fig. S1) were generated form the map of Land Development Department^66^.

**Table 2:** Environmental factors, data source, format data, and data scale to generated species distribution models in the eastern part of Thailand.

Environmental factor	Data source	Format data	Data scale
Distance to permanent water sources (km)	https://data.go.th/en/dataset/landuse	Shapefile	1:50,000
Distance to reservoirs (km)	https://data.go.th/en/dataset/landuse	Shapefile	1:50,000
Distance to land use types (km)	https://data.go.th/en/dataset/landuse	Shapefile	1:50,000
Distance to aquaculture farms (km)	https://data.go.th/en/dataset/landuse	Shapefile	1:50,000
Distance to permanent streams (km)	https://data.go.th/en/dataset/landuse	Shapefile	1:50,000
Distance to temporary streams (km)	https://data.go.th/en/dataset/landuse	Shapefile	1:50,000
Slopes (%)	https://data.go.th/en/dataset/landuse	Shapefile	1:50,000
Altitude above sea level (asl, m)	https://data.go.th/en/dataset/landuse	Shapefile	1:50,000

Fawcett^67^ has explained the interpretation of model analysis results from MaxEnt by considering the area under the Receiver Operating Characteristic (ROC) curve, also known as the Area Under the ROC Curve (AUC). The AUC can be divided into five levels as follows: AUC = 0–0.2 is unsuited; > 0.2–0.4 is poorly suited; > 0.4–0.6 is moderately suited; > 0.6–0.8 is well suited; > 0.8–1.0 is very well suited. The accuracy of SDMs was assessed by used prevalence, kappa and the true skill statistic (TSS)^68^.

### Analysis

One-way ANOVA was used to test significant differences in crop raiding among provinces in the eastern part of Thailand among the years 2000, 2010, and 2020 at *p*-value ≤ 0.05. The relative frequencies were the percentage of households in each year divided by total percentage of households in each province.

### Ethics approval

The study was conducted in accordance with the Declaration of Helsinki and approved by the Institutional Review Board of Mahidol University—Institute Animal Care and Use Committee (MU—CIRB 2020/123.2605, 4 June 2020). The animal study protocol was approved by the Institutional Review Board of Mahidol University—Institute Animal Care and Use Committee (MU—IACUC No. F02‐63‐004, 1 May 2020). 

### Consent to participate

Accordance statement for plants, stating that "all the procedure included in the study is in accordance with the relevant guideline", and informed consent was obtained from all subjects and/or their legal guardian(s).

### Supplementary Information

Supplementary Information.

## Data Availability

Data will be made available in a repository such as Dryad upon final acceptance. For review purposes, the raw data files can be accessed via the temporary link: https://drive.google.com/file/d/1RVtqWWLqmx-GtbmTuFZ2Ze91ur-JZV5n/view?usp=share_link.
