# Supplementary material for: Environmental factors induced crop raiding by wild Asian elephant (Elephas maximus) in the Eastern Economic Corridor, Thailand
Source: Sci Rep. 2023 Aug 17;13:13388. doi: 10.1038/s41598-023-40070-3 (PMC10435484; doi:10.1038/s41598-023-40070-3)
Supplement: Supplementary file 1 — Supplementary Information. [file 41598_2023_40070_MOESM1_ESM.pdf]

Environmental factors induced crop raiding by wild Asian elephant (*Elephas maximus*) in the Eastern Economic Corridor, Thailand

Maneepailin Wettasin[1], Rattanawat Chaiyarat[2,*], Namphung Youngpoy[2], Navee Cheachean[2] Ronglarp Sukmasuang[3] and Phanwimol Tanhan[4]

Supplementary Table S1. Estimates of relative contributions of the environmental variables, the overall prevalence of training curve of the receiver operating characteristic (ROC) curve and area under the ROC (AUC), test gain, and test AUC of the MaxEnt model in 2020 (n = 10,196 points), 2010 (n = 89 points) and 2000 (n = 54 points).

**Environmental variable**	**Abbreviation**	**Average contribution**			**Average permutation**			**AUC**		
		**2020**	**2010**	**2000**	**2020**	**2010**	**2000**	**2000**	**2010**	**2000**
Elevation (asl, m)	elevation	15.8	15.8	18.9	19.8	19.8	17.2	0.6	0.6	0.6
Land use	landuse	30.9	30.9	17.7	19.2	19.2	17.1	0.7	0.7	0.6
Distance from permanent river (m)	permanentriver	7.1	7.1	10.2	8	8	7.5	0.6	0.6	0.6
Distance from permanent stream (m)	permanentstream	2.8	2.8	4.4	3.4	3.4	4.4	0.5	0.5	0.5
Distance from permanent water source (m)	permanentwatersource	32.9	32.9	25.1	39.7	39.7	28.8	0.6	0.6	0.6
Distance from reservoir (m)	reservoir	6.4	6.4	16.7	6.9	6.9	17.5	0.6	0.6	0.6
Slope (%)	slope	4.2	4.2	6.9	3	3	7.6	0.6	0.6	0.6

Supplementary Table S2. Estimates of relative contributions of the environmental variables, overall prevalence of training curve of the receiver operating characteristic (ROC) curve and area under the ROC (AUC), test gain and test AUC, Kappa, Sensitivity, Specificity and true skill statistic (TSS) value of the MaxEnt model in 2000, 2010, and 2020 (Training samples = 198, Test sample = 65).

**Year**	**Ele-phant**	**Regularized training gain**	**Unregu-larized training gain**	**Itera-tions**	**Training AUC**	**Test gain**	**Test AUC**	**AUC Standard Deviation**	**Kappa**	**Sensi-tivity**	**Speci-ficity**	**TSS**
2020	0	0.577	0.783	800	0.822	0.61	0.783	0.022	0.029	0.962	0.395	0.357
	1	0.632	0.884	680	0.843	0.414	0.739	0.028	0.015	0.992	0.234	0.226
	2	0.594	0.813	840	0.829	0.433	0.74	0.028	0.02	0.962	0.315	0.277
	3	0.572	0.803	680	0.831	0.608	0.775	0.028	0.026	0.966	0.37	0.336
	4	0.584	0.802	720	0.83	0.544	0.773	0.023	0.017	0.992	0.264	0.257
	5	0.614	0.806	560	0.83	0.387	0.735	0.027	0.021	0.981	0.307	0.288

	6	0.623	0.837	1,040	0.833	0.401	0.743	0.029	0.022	0.969	0.332	0.301
	7	0.563	0.774	960	0.821	0.588	0.782	0.026	0.016	1	0.239	0.239
	8	0.593	0.785	740	0.825	0.508	0.777	0.025	0.004	1	0.074	0.074
	9	0.568	0.785	680	0.824	0.584	0.782	0.023	0.024	0.977	0.337	0.314
	10	0.561	0.765	740	0.826	0.608	0.776	0.027	0.016	0.981	0.26	0.241
	11	0.574	0.792	1,100	0.829	0.557	0.777	0.026	0.021	0.977	0.312	0.289
	12	0.598	0.836	800	0.837	0.476	0.758	0.029	0.013	0.992	0.211	0.203
	13	0.543	0.775	620	0.83	0.692	0.806	0.027	0.014	0.992	0.227	0.219
	14	0.612	0.824	680	0.833	0.404	0.743	0.029	0.019	0.992	0.279	0.271
	Average	0.587	0.804	776	0.829	0.521	0.766	0.026	0.019	0.982	0.277	0.259
2010	0	0.581	0.737	700	0.584	0.774	0.027	0.584	0.038	0.927	0.434	0.362
	1	0.585	0.757	1,160	0.561	0.773	0.024	0.561	0.016	0.987	0.218	0.206
	2	0.565	0.727	820	0.567	0.776	0.024	0.567	0.023	0.981	0.293	0.274
	3	0.589	0.748	920	0.538	0.776	0.026	0.538	0.033	0.95	0.392	0.342
	4	0.604	0.775	1,720	0.474	0.762	0.025	0.474	0.022	0.984	0.282	0.266
	5	0.624	0.793	1,160	0.348	0.728	0.026	0.348	0.025	0.965	0.318	0.284
	6	0.573	0.723	960	0.575	0.765	0.025	0.575	0.023	0.987	0.29	0.277
	7	0.572	0.739	1,120	0.522	0.77	0.027	0.522	0.019	0.987	0.253	0.241
	8	0.607	0.739	820	0.497	0.763	0.024	0.497	0.004	0.997	0.069	0.066
	9	0.556	0.711	860	0.632	0.797	0.021	0.632	0.029	0.981	0.338	0.319
	10	0.633	0.796	800	0.346	0.727	0.026	0.346	0.021	0.981	0.276	0.257
	11	0.585	0.754	1,040	0.555	0.764	0.026	0.555	0.026	0.962	0.331	0.293
	12	0.61	0.767	860	0.432	0.747	0.025	0.432	0.013	0.994	0.179	0.172
	13	0.611	0.761	1,220	0.437	0.739	0.025	0.437	0.019	0.991	0.245	0.235
	14	0.571	0.715	1,180	0.563	0.771	0.023	0.563	0.016	0.991	0.219	0.209
	Average	0.591	0.75	1,022.7	0.509	0.762	0.025	0.509	0.022	0.978	0.276	0.254
2000	0	0.413	0.641	800	0.8	0.36	0.731	0.024	0.021	0.996	0.294	0.291
	1	0.44	0.656	680	0.812	0.199	0.691	0.03	0.01	0.989	0.176	0.165
	2	0.409	0.648	600	0.802	0.422	0.747	0.026	0.014	1	0.22	0.22
	3	0.366	0.545	620	0.782	0.51	0.771	0.024	0.017	0.996	0.251	0.247
	4	0.422	0.644	740	0.807	0.334	0.72	0.029	0.013	0.992	0.208	0.2
	5	0.4	0.612	780	0.801	0.385	0.741	0.03	0.013	0.989	0.212	0.201
	6	0.394	0.597	680	0.79	0.425	0.751	0.024	0.013	1	0.207	0.207
	7	0.409	0.6	680	0.797	0.38	0.73	0.03	0.01	0.985	0.181	0.166
	8	0.482	0.723	1,080	0.821	0.135	0.677	0.028	0.004	0.996	0.072	0.068
	9	0.412	0.653	680	0.805	0.306	0.718	0.027	0.018	0.989	0.269	0.257
	10	0.447	0.645	640	0.799	0.272	0.711	0.026	0.012	0.992	0.199	0.192
	11	0.43	0.651	680	0.806	0.306	0.709	0.027	0.016	0.992	0.247	0.239
	12	0.442	0.632	780	0.801	0.233	0.698	0.027	0.008	0.992	0.143	0.135

	13	0.423	0.629	700	0.799	0.286	0.702	0.029	0.011	0.992	0.187	0.18
	14	0.426	0.645	800	0.799	0.355	0.731	0.025	0.011	0.996	0.184	0.18
	Average	0.421	0.635	729.3	0.801	0.327	0.722	0.027	0.013	0.993	0.203	0.196

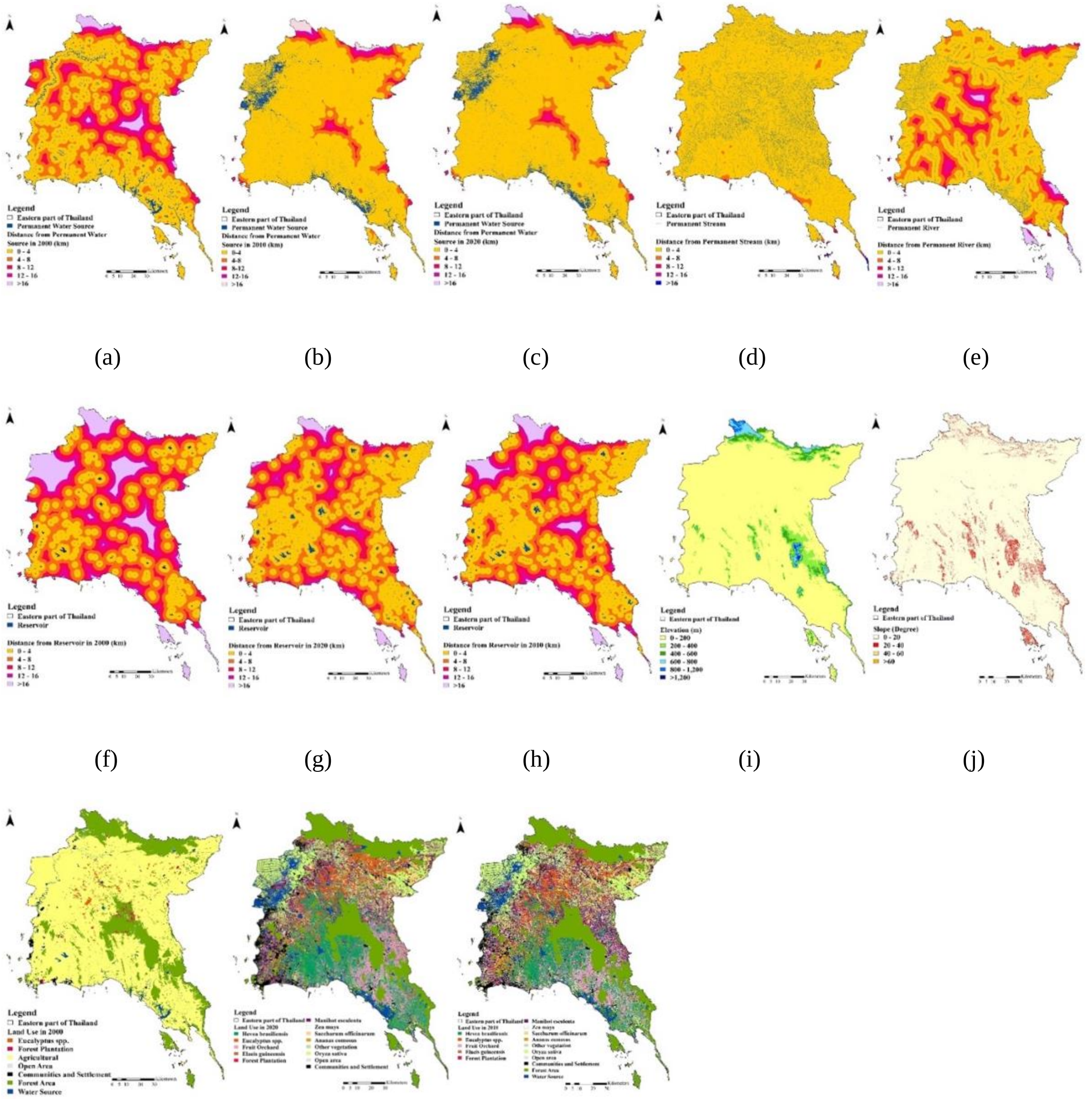

(a) (b) (c) (d) (e)

(f) (g) (h) (i) (j)

(k) (l) (m)

Supplementary Fig. S1. Environmental factors used to analyze species distribution models (SDMs) of wild Asian elephant in the eastern part of Thailand: Distance from Permanent Water Source (km) in (a) 2000, (b) 2010, (c) 2020, (d) Distance from Permanent Stream (km), (e) Distance from Permanent River (km), Distance from Reservoir (km) in (f) 2000, (g) 2010, (h) 2020, (i) Elevation (m), (j) Slope (%), Land Use in (k) 2000, (l) 2010, and (m) 2020.
